# Application of single step genomic BLUP under different uncertain paternity scenarios using simulated data

**DOI:** 10.1371/journal.pone.0181752

**Published:** 2017-09-28

**Authors:** Rafael Lara Tonussi, Rafael Medeiros de Oliveira Silva, Ana Fabrícia Braga Magalhães, Rafael Espigolan, Elisa Peripolli, Bianca Ferreira Olivieri, Fabieli Loise Braga Feitosa, Marcos Vinicíus Antunes Lemos, Mariana Piatto Berton, Hermenegildo Lucas Justino Chiaia, Angelica Simone Cravo Pereira, Raysildo Barbosa Lôbo, Luiz Antônio Framartino Bezerra, Cláudio de Ulhoa Magnabosco, Daniela Andressa Lino Lourenço, Ignácio Aguilar, Fernando Baldi

**Affiliations:** 1 Department of Animal Science, School of Agricultural and Veterinarian Sciences, Jaboticabal, São Paulo, Brazil; 2 Department of Nutrition and Animal Production, Faculty of Animal Science and Food Engineering, Pirassununga, Brazil; 3 National Association of Breeders and Researchers (ANCP), Ribeirão Preto, Brazil; 4 Department of Genetic, Medical School of Ribeirão Preto, Ribeirão Preto, Brazil; 5 Brazilian Agricultural Research Corporation (EMBRAPA), Distrito Federal, Brazil; 6 Department of Animal and Dairy Science, University of Georgia, Athens, Georgia, United States of America; 7 Department of Animal Breeding, National Institute of Agricultural Research, Las Brujas, Uruguay; University of Illinois, UNITED STATES of America

## Abstract

The objective of this study was to investigate the application of BLUP and single step genomic BLUP (ssGBLUP) models in different scenarios of paternity uncertainty with different strategies of scaling the G matrix to match the A22 matrix, using simulated data for beef cattle. Genotypes, pedigree, and phenotypes for age at first calving (AFC) and weight at 550 days (W550) were simulated using heritabilities based on real data (0.12 for AFC and 0.34 for W550). Paternity uncertainty scenarios using 0, 25, 50, 75, and 100% of multiple sires (MS) were studied. The simulated genome had a total length of 2,333 cM, containing 735,293 biallelic markers and 7,000 QTLs randomly distributed over the 29 BTA. It was assumed that QTLs explained 100% of the genetic variance. For QTL, the amount of alleles per loci randomly ranged from two to four. The BLUP model that considers phenotypic and pedigree data, and the ssGBLUP model that combines phenotypic, pedigree and genomic information were used for genetic evaluations. Four ways of scaling the mean of the genomic matrix (**G**) to match to the mean of the pedigree relationship matrix among genotyped animals (**A**_22_) were tested. Accuracy, bias, and inflation were investigated for five groups of animals: ALL = all animals; BULL = only bulls; GEN = genotyped animals; FEM = females; and YOUNG = young males. With the BLUP model, the accuracies of genetic evaluations decreased for both traits as the proportion of unknown sires in the population increased. The EBV accuracy reduction was higher for GEN and YOUNG groups. By analyzing the scenarios for YOUNG (from 0 to 100% of MS), the decrease was 87.8 and 86% for AFC and W550, respectively. When applying the ssGBLUP model, the accuracies of genetic evaluation also decreased as the MS in the pedigree for both traits increased. However, the accuracy reduction was less than those observed for BLUP model. Using the same comparison (scenario 0 to 100% of MS), the accuracies reductions were 38 and 44.6% for AFC and W550, respectively. There were no differences between the strategies for scaling the **G** matrix for ALL, BULL, and FEM groups under the different scenarios with missing pedigree. These results pointed out that the uninformative part of the **A**_**22**_ matrix and genotyped animals with paternity uncertainty did not influence the scaling of **G** matrix. On the basis of the results, it is important to have a **G** matrix in the same scale of the **A**_**22**_ matrix, especially for the evaluation of young animals in situations with missing pedigree information. In these situations, the ssGBLUP model is an appropriate alternative to obtain a more reliable and less biased estimate of breeding values, especially for young animals with few or no phenotypic records. For accurate and unbiased genomic predictions with ssGBLUP, it is necessary to assure that the **G** matrix is compatible with the **A**_22_ matrix, even in situations with paternity uncertainty.

## Introduction

The multiple sires natural mating is the most common mating system in extensive beef cow-calf production, where several sires are kept in the same paddock to breed with cows during the mating season. Despite the management advantages of this mating system, it does not allow the progeny paternity identification, increasing the occurrence of missing pedigree and, consequently, compromising the genetic evaluation reliability.

Several methods have been suggested to increase the genetic evaluation reliability of animals with paternity uncertainty. In this regard, the unknown parent groups (UPG) can be included in the mixed model equations to account for genetic differences among defined animal groups in genetic evaluations [1]. Henderson [2] and Kennedy [3] noticed that ignoring genetic groups or defining poor genetic groups could introduce bias in genetic evaluations. Westell et al. [4] developed rules to setting up genetic groups in mixed model equations. The hierarchical animal model (HIER) proposed by Cardoso and Tempelman [5] is another procedure to perform genetic evaluation of animals with paternity uncertainty. This method combines phenotypic records and *a priori* information to deduce the *a posteriori* probabilities of the candidate sire, inferring the animal genetic merit with paternity uncertainty and its respective sire. Furthermore, the DNA markers can be used to assign calves to their individual sires based on inheritance rules. However, in most of the extensive beef cow-calf production systems, it is not common to identify or record the sire or group of sires used in the mating season, since the sires are only used in one mating season (cleanup bulls).

Recently, Legarra et al. [6], Misztal et al. [7] and Aguilar [8] proposed the single-step genomic BLUP procedure (ssGBLUP). This procedure combines the pedigree-based relationship matrix with the genomic relationship matrix into a single matrix (**H**) to predict the genomic estimated breeding value (GEBV). Several studies have reported that the ssGBLUP is computationally efficient and accurate for genomic evaluation purposes [8–11]. The pedigree-based relationship matrix of genotyped animals (**A**_22_ matrix) and the genomic relationship matrix (**G** matrix) should be compatible in order to decrease the occurrence of biased genetic parameter estimates [11, 12, 13, 14]. Hence, the **G** matrix is adjusted to reduce the differences between the average diagonal and the average off-diagonal elements in **G** and **A**_22_ matrices. Chen et al. [12] reported that the scale of **G** influenced the ranking of genotyped versus non-genotyped animals. Vitezica et al. [14] by exploiting the ssGBLUP, derived a formal proof and showed that a well-constructed **G** provides more accurate and less biased GEBV than a multistep approach. Up to date, there are no reports about the consequences of using the ssGBLUP in situations with the multiple sires natural mating system, where the **A**_22_ matrix is less informative due to the presence of missing pedigree. In this situation, problems of compatibility and scaling between **A**_22_ and **G** matrices are expected, affecting the reliability of genetic evaluations. In this regard, Forni et al. [13] reported overestimated variances and biased GEBV when the **A**_22_ matrix was sparser than the **G** matrix.

The ssGBLUP procedure was developed to be implemented in situations without missing pedigree or informative **A**_22_ matrix. Some problems have been reported when the ssGBLUP model included UPGs [15]. Misztal et al. [15] worked with missing pedigrees and studied several options to include UPG in the ssGBLUP model to eliminate or reduce biases in the UPG solutions and in the animal predictions. The authors explained that potential bias could happen in genomic EBV using ssGBLUP with UPG. Recently, Tsuruta et al. [1] examined how to define the UPG assigned in mixed-model equations to reduce bias and increase accuracy in genomic evaluations for young Holstein bulls using ssGBLUP model.

In Brazil, there are many herds belonging to breeding programs that frequently use the multiple sires natural mating system, and it is common observe roughly 60% of the progeny with unknown sires. Additionally, there is a growing interest in commercial beef cattle herds lacking the pedigree structure to run genetic evaluations with the ssGBLUP so as to identify and commercialize animals with genetic evaluation information. In this context, it is important to evaluate the technical feasibility of the ssGBLUP in situations with paternity uncertainty. There are many doubts and concerns about the most adequate **G** matrix scaling method under paternity uncertainty scenarios and, their impact upon genomic evaluation (accuracy and bias). Currently, models that consider paternity uncertainty using genomic information are not used in animal breeding programs. Therefore, the objective of this study was to investigate the application of BLUP and ssGBLUP models under different scenarios of paternity uncertainty with different strategies of scaling the **G** matrix to match the **A**_22_ matrix, using simulated data for beef cattle.

## Materials and methods

Phenotypes, pedigree, and genotypes were simulated using the software QMSim version 1.00 [16]. Two traits assuming low and moderate heritabilities were simulated: age at first calving (AFC; h^2^ = 0.12) and weight at 550 days (W550; h^2^ = 0.34). Heritabilities were based on real data estimates [17–19], and the phenotypic variance was assumed to be 1.0. Ten replicates were performed for each trait and results were averaged among replicates.

### Simulated population

A historical population was created from generation zero to 2,020, with a constant size of 2,000 animals (from generation zero to 1,000) to generate different levels of linkage disequilibrium (LD). A gradual reduction in the number of animals (from 2,000 to 600) produced a “bottleneck effect” and consequently, genetic drift and LD from generation 1,001 to 2,020.

Two hundred out of the 600 animals from the latest generation of the historical population were selected (males and females equally distributed) for the expanded population, which had its effective size simulated based on the real population [20]. To simulate the expanded population, a mating system based on a random union of gametes, an absence of selection, an exponential growth of the number of females, and an average of five progeny per dam were considered.

After the expansion process, 240 males and 6,000 females from the last generation were randomly selected, including the founder animals from the selection population. This population was spanned over 10 generations and the selected males and females from each generation were randomly mated, generating a single progeny with equal probability of being a male or a female. The replacement rate of sires and dams was kept constant over the generations at a rate of 20% and 60%, respectively. The genotypes of 10,000 animals of the last three generations (8, 9, and 10) were randomly selected. The estimated LD between adjacent markers in the 8, 9 and 10 generations were 0.17, 0.18 and 0.18, respectively. These results were similar to those reported by Espigolan et al. [21] using a Nellore cattle population genotyped with the BovineHD BeadChip (Illumina).

### Simulated genome

The simulated genome had a total length of 2,333 cM, 735,293 markers and 7,000 QTLs randomly distributed over the 29 *Bos Taurus* autosomes (BTA). The length of the bovine genome was based on Base_4.6.1 [22] and it was assumed that QTLs explained 100% of the genetic variance.

The number of markers and QTLs per chromosome ranged from 12,931 to 46,495 and from 121 to 438, respectively. All markers were bi-allelic, mimicking SNPs present in the bovine commercial panels. For QTLs, the amount of alleles per loci randomly ranged from two to four. Minor allele frequencies (MAF) were assumed equally for markers and QTLs alleles. QTLs allele effects were sampled from a gamma distribution with a shape parameter equal to 0.4 [23].

A mutation rate of 10^−5^ for markers and QTLs in the historical populations was considered. A total of 335,000 markers (with MAF greater or equal to 0.02) and 1,000 QTLs were randomly selected from the last generation of the historical population to generate genotypic data for the selection population. The animal phenotypes were computed as the sum of the QTLs effects and an error term sampled from a normal distribution with zero mean and variance equal to 0.88 for AFC and 0.66 for W550.

### BLUP and ssGBLUP models

In the BLUP model, a traditional genetic evaluation was performed using pedigree and phenotypic information. The model can be represented as follows:

Y=Xb+Zu+e

where ***y*** is the vector of phenotype, ***b*** is the vector of fixed effects, ***u*** is the vector of additive genetic effects, ***X*** e ***Z*** are incidence matrices and ***e*** is the vector of random residuals. Considering an infinitesimal model, $var(u)=A\sigma_{u}^{2}$, where **A** is the numerator relationship matrix obtained from pedigree information and $\sigma_{u}^{2}$ is the variance of genetic effect.

In the single-step genomic BLUP (ssGBLUP) proposed by Misztal et al. [7], the inverse of the numerator relationship matrix (**A**^-1^) was replaced by **H**^**-1**^ that combines pedigree and genomic information. The **H**^**-1**^ was constructed according Aguilar et al. [8] as showed below:

$$H^{-1}{=A}^{-1}+\left[\begin{array}{cc} 0 & 0\\ 0 & G^{-1}-A_{22}^{-1} \end{array}\right],$$

where **H**^**-1**^ is the inverse of the realized relationship matrix that incorporates the inverse of the genomic relationship matrix (**G**^**-1**^**)** and the inverse of the numerator relationship matrix of genotyped animals $A_{22}^{-1}$. The **G** matrix was created according to VanRaden [24]:

$$G=\frac{(M-P)(M-P)\prime }{2\sum_{j=1}^{m}p_{j}(1-p_{j})}$$

where **M** is a matrix of marker alleles with *m* columns (*m* = total number of markers) and *n* rows (*n* = total number of genotyped individuals), and **P** is a matrix containing the frequency of the second allele (p_j_), expressed as 2p_j_. **M**_ij_ was 0 if the genotype of individual *i* for SNP *j* was homozygous for the first allele, 1 if heterozygous, or 2 if the genotype was homozygous for the second allele.

### Scenarios

A total of 25 scenarios were tested for each trait (50 scenarios in total) with BLUP and ssGBLUP models, considering five proportions of paternity uncertainty and four strategies of scaling the **G** matrix to match to the **A**_22_ matrix_._ These scenarios were proposed in order to evaluate the impact of disinformation (missing pedigree) in the **A**_22_ matrix on the scale adjustment of the **G** matrix. In this sense, the **A** matrix was created assuming different proportions of multiple sires (0, 25, 50, 75, and 100%) in the genotyped animals. To evaluate the impact of scaling the **G** matrix on **A**_22_ matrix under different situations with missing pedigree, four strategies of scaling the **G** matrix to match to the **A**_22_ matrix were tested: S_1_—considering mean diagonal **A**_22_ = mean diagonal **G** and mean-off diagonal **A**_22_ = mean-off diagonal **G** matrix, this is the default option in the preGSf90 program to reduce the differences between the average diagonal and the average off-diagonal elements in the **G** and **A**_22_ matrices [12]; S_2_—no scaling between the **A**_22_ and **G** matrices, the elements of the **G** matrix were not adjusted for the elements of the **A**_22_ matrix; S_3_—scaling only the animals which have known sire and dam, the elements of the **G** matrix were adjusted only considering the elements of the **A**_22_ matrix of animals with both parents known; and S_4_—scaling only those animals which have one known parent, the elements of the **G** matrix were adjusted only considering the elements of the **A**_22_ matrix of animals with at least one parent known.

For each scenario, the accuracy of prediction, bias, and inflation were calculated for five groups of animals: ALL = all animals (66,240); BULL = only bulls with at least one progeny (1,563); GEN = genotyped animals (10,000); FEM = females (36,346); and YOUNG = young males without progeny (5,803). The accuracy of prediction was computed as the correlation between the true breeding value (TBV) and EBV or genomic EBV (GEBV). Bias was measured as the difference between predicted and simulated breeding values of the candidates [14]. Regression of TBV on EBV was used as a measure of the inflation of the prediction method, where a regression coefficient equal to one denotes no inflation. Results were the mean of 10 replicates of each scenario. For AFC, a total of 10,000; 91; 10,000; 5,008 and 4,901 genotyped animals for ALL, BULL, GEN, FEM, and YOUNG were used, respectively. For W550, a total of 10,000; 98; 10,000; 5,012 and 4,890 genotyped animals for ALL, BULL, GEN, FEM, and YOUNG were used, respectively. The variance component estimation and solutions were obtained by BLUPF90 family programs [25–26].

## Results and discussion

The variance component estimates obtained for AFC and W550 with the traditional pedigree (REML) and genomic information (GREML) with different proportions of MS are presented in Table 1. It is important to highlight that the software used for the simulation did not estimate the variance components, besides, it uses heritability values provided by the user (0.12 for AFC and 0.34 for W550).

**Table 1:** Estimation of variance components, heritability and standard errors for AFC and W550 using traditional and genomic REML with different proportions of multiple sires.

Trait	MS	REML			GREML		
		$\sigma_{a}^{2}$	$\sigma_{e}^{2}$	h^2^±SE	$\sigma_{a}^{2}$	$\sigma_{r}^{2}$	h^2^±SE
	0%	0.13±0.004	0.88±0.002	0.13±0.003	0.10±0.002	0.89±0.003	0.10±0.002
	25%	0.14±0.004	0.88±0.002	0.14±0.004	0.13±0.004	0.87±0.003	0.13±0.003
AFC (true h^2^: 012)	50%	0.16±0.006	0.88±0.002	0.15±0.005	0.16±0.005	0.86±0.003	0.15±0.004
	75%	0.18±0.007	0.87±0.003	0.17±0.006	0.18±0.006	0.86±0.003	0.17±0.005
	100%	0.20±0.009	0.86±0.003	0.19±0.007	0.17±0.005	0.88±0.003	0.16±0.004
	0%	0.35±0.008	0.66±0.002	0.35±0.006	0.31±0.008	0.67±0.001	0.32±0.006
	25%	0.41±0.012	0.66±0.002	0.38±0.007	0.40±0.001	0.64±0.002	0.39±0.007
W550 (true h^2^: 0.34)	50%	0.48±0.014	0.67±0.002	0.42±0.008	0.49±0.014	0.63±0.002	0.44±0.007
	75%	0.55±0.017	0.64±0.002	0.46±0.008	0.55±0.015	0.63±0.002	0.46±0.007
	100%	0.63±0.020	0.62±0.003	0.50±0.009	0.55±0.017	0.68±0.002	0.45±0.008

AFC = age at first calving; W550 = weight at 550 days; MS = percentage of multiple sires; REML = restricted maximum likelihood estimation; GREML = genomic restricted maximum likelihood estimation $\sigma_{a}^{2}$ = additive genetic variance; $\sigma_{e}^{2}$ = residual variance; h^2^ = heritability; SE = standard errors

In the BLUP model, the additive genetic variance ranged from 0.13 to 0.20 and from 0.35 to 0.63 for AFC and W550, respectively. For both traits, the highest additive genetic variance was observed for the scenario with 100% of MS. The additive genetic variances were overestimated as the percentage of MS increased in the population, being more noticeable for the trait with a moderate heritability (W550). Considering 50% of MS, the additive genetic variance increased 18.75 and 27% for AFC and W550, respectively, when compared to the scenario with 0% of MS. Nevertheless, with 100% of MS, this increase was 35 and 44.4%, respectively. These results can be explained by the reduction in the number of inbred animals as the proportion of MS increased in the pedigree (Table 2). The increase in the proportion of MS may have led to the increase in the additive genetic variance between families since the families were less related due to missing pedigree.

**Table 2:** Pedigree structure with different proportions of multiple sires.

	Percentage of multiple sires				
	0%	25%	50%	75%	100%
All animals	66,240	66,240	66,240	66,240	66,240
Inbred animals for AFC	23,326	20,927	18,534	16,153	13,785
Inbred animals for W550	19,134	16,830	14,542	12,292	10,062
Bulls	1,536	1,536	1,536	1,534	1,297
Dams	16,800	16,800	16,800	16,800	16,800
Progeny only known sire	0	0	0	0	0
Progeny only known dam	0	2,500	5,000	7,500	10,000
Progeny known sire and dam	60,000	57,500	55,000	52,500	50,000

In addition, the poor data structure that did not match to the animal model to estimate the variance components might be another reason that led to the additive genetic overestimation. Nietlisbach et al. [27] stated that the inbreeding effect on the heritability estimate depends on the considered population, but increasing the level of inbreeding in the population reduced the heritability estimates. When applying the ssGBLUP model for the same scenarios (0 to 50% and 0 to 100% of MS) the additive genetic variance increased 37.5 and 41.2% for AFC, and 36.7 and 43.6% for W550, respectively. The inclusion of the genomic relationship matrix decreased the additive genetic variance estimates for both traits in all evaluated scenarios (Table 2).

Accuracies of genetic evaluation and bias for all studied groups using BLUP and ssGBLUP models are shown in Tables 3 and 4. With the BLUP model, the accuracies of genetic evaluations decreased for both traits as the proportion of unknown sires in the population increased. The EBV accuracy reduction was higher for GEN and YOUNG groups. Therefore, comparing the scenarios for YOUNG group (from 0 to 100% of MS) the decrease was 87.8 and 86% for AFC and W550, respectively. These results pointed out that in situations of missing pedigree, the selection of young animals with EBV estimated by BLUP can be really unreliable.

**Table 3:** Breeding value accuracy estimates and standard errors for AFC and W550 using BLUP and ssGBLUP models with different proportions of multiple sires.

			Percentage of multiple sires				
			0%	25%	50%	75%	100%
AFC	ALL	BLUP	0.79±0.006	0.76±0.005	0.73±0.005	0.70±0.005	0.68±0.004
		ssGBLUP	0.80±0.006	0.78±0.005	0.76±0.005	0.73±0.005	0.69±0.004
	BULL	BLUP	0.87±0.005	0.86±0.005	0.84±0.006	0.83±0.006	0.85±0.005
		ssGBLUP	0.87±0.005	0.85±0.005	0.81±0.004	0.79±0.004	0.85±0.005
	GEN	BLUP	0.46±0.007	0.20±0.005	0.16±0.004	0.16±0.005	0.30±0.006
		ssGBLUP	0.55±0.009	0.57±0.009	0.56±0.009	0.56±0.009	0.56±0.010
	FEM	BLUP	0.81±0.005	0.78±0.005	0.76±0.005	0.74±0.005	0.72±0.004
		ssGBLUP	0.81±0.005	0.80±0.005	0.78±0.005	0.76±0.005	0.73±0.004
	YOUNG	BLUP	0.41±0.007	0.14±0.005	0.08±0.005	0.06±0.007	0.05±0.008
		ssGBLUP	0.50±0.009	0.50±0.008	0.46±0.008	0.42±0.007	0.31±0.007
W550	ALL	BLUP	0.92±0.001	0.89±0.001	0.86±0.002	0.84±0.02	0.82±0.002
		ssGBLUP	0.92±0.001	0.92±0.001	0.90±0.001	0.88±0.001	0.84±0.001
	BULL	BLUP	0.97±0.001	0.96±0.001	0.95±0.001	0.93±0.001	0.97±0.001
		ssGBLUP	0.97±0.001	0.97±0.001	0.95±0.001	0.94±0.001	0.97±0.001
	GEN	BLUP	0.60±0.005	0.29±0.003	0.25±0.003	0.28±0.003	0.41±0.003
		ssGBLUP	0.72±0.006	0.70±0.005	0.68±0.004	0.67±0.004	0.66±0.004
	FEM	BLUP	0.93±0.001	0.91±0.001	0.89±0.001	0.88±0.001	0.87±0.002
		ssGBLUP	0.93±0.001	0.92±0.001	0.91±0.001	0.89±0.001	0.87±0.002
	YOUNG	BLUP	0.50±0.004	0.16±0.003	0.09±0.005	0.06±0.005	0.07±0.006
		ssGBLUP	0.65±0.006	0.60±0.005	0.53±0.003	0.46±0.003	0.36±0.003

AFC = age at first calving; W550 = weight at 550 days; ALL = all animals; BULL = bulls; GEN = genotyped animals; FEM = females; YOUNG = young males; BLUP = best linear unbiased prediction; ssGBLUP = single step genomic BLUP

**Table 4:** Bias and standard errors using BLUP and ssGBLUP with different proportions of multiple sires.

			Percentage of multiple sires				
			0%	25%	50%	75%	100%
AFC	ALL	BLUP	1.00±0.005	1.01±0.006	1.01±0.007	1.02±0.007	1.02±0.007
		ssGBLUP	0.95±0.005	0.98±0.005	0.99±0.006	1.01±0.006	0.91±0.006
	BULL	BLUP	1.01±0.005	0.99±0.006	0.97±0.007	0.95±0.008	0.93±0.006
		ssGBLUP	1.01±0.006	1.04±0.006	1.04±0.007	1.03±0.007	0.93±0.007
	GEN	BLUP	1.03±0.014	0.24±0.006	0.18±0.003	0.21±0.005	0.78±0.013
		ssGBLUP	0.94±0.020	0.85±0.015	0.80±0.013	0.81±0.012	0.91±0.017
	FEM	BLUP	1.01±0.005	1.02±0.006	1.04±0.007	1.05±0.007	1.07±0.007
		ssGBLUP	0.95±0.005	0.96±0.006	0.97±0.006	0.10±0.007	1.01±0.007
	YOUNG	BLUP	1.03±0.018	0.18±0.006	0.09±0.006	0.07±0.009	0.08±0.012
		ssGBLUP	0.91±0.020	0.81±0.015	0.68±0.013	0.58±0.009	0.38±0.009
W550	ALL	BLUP	1.00±0.002	1.01±0.003	1.02±0.002	1.04±0.002	1.07±0.003
		ssGBLUP	0.99±0.002	1.03±0.003	1.06±0.003	1.07±0.003	1.07±0.003
	BULL	BLUP	1.01±0.002	1.00±0.002	0.98±0.002	0.97±0.002	0.94±0.003
		ssGBLUP	1.03±0.002	1.06±0.002	1.08±0.002	1.08±0.003	0.95±0.003
	GEN	BLUP	0.97±0.009	0.26±0.002	0.21±0.003	0.26±0.003	0.60±0.005
		ssGBLUP	1.06±0.009	0.89±0.006	0.75±0.005	0.73±0.006	0.78±0.006
	FEM	BLUP	1.00±0.003	1.02±0.003	1.05±0.003	1.07±0.003	1.11±0.003
		ssGBLUP	0.99±0.003	1.01±0.002	1.04±0.003	1.06±0.004	1.08±0.004
	YOUNG	BLUP	0.97±0.006	0.14±0.003	0.07±0.004	0.05±0.004	0.07±0.007
		ssGBLUP	1.05±0.009	0.80±0.006	0.60±0.005	0.48±0.003	0.34±0.004

AFC = age at first calving; W550 = weight at 550 days; ALL = all animals; BULL = bulls; GEN = genotyped animals; FEM = females; YOUNG = young males; BLUP = best linear unbiased prediction; ssGBLUP = single step genomic BLUP

By their apply the ssGBLUP model, the accuracies of the genetic evaluation also decreased when increasing the proportion of MS in the pedigree, observed for both traits. However, the reduction in the accuracies was less evident than those observed for the BLUP model. Using the same comparison for YOUNG group (scenario 0 to 100% of MS), the reduction in the accuracies was 38 and 44.6% for AFC and W550, respectively. These observations may also support the argument that the ssGBLUP model can be more accurate for genetic evaluation of young animals in situations with missing pedigree records.

The accuracies for ALL, BULL, and FEM groups were similar for BLUP and ssGBLUP models for both traits. Additionally, the EBV and GEBV accuracies decreased as the proportion of MS increased. It is expected that genomic information would contribute less for a group of animals that have enough phenotypic or progeny information contributing to the accuracy [28]. This results support a previous study on real pig data by Forni et al. [13] that showed that the inclusion of the **G** matrix had little impact on the accuracy of sires, but higher impact on the accuracy of females with few phenotypic records.

Considering the scenario with 0% of MS for both traits, the accuracies of genetic evaluation for ALL, BULL, and FEM groups remained almost constant with the inclusion of the **G** matrix in the population. However, the GEBV obtained with the ssGBLUP model for YOUNG group increased 22 and 30% for AFC and W550, respectively. Wiggans et al. [29] reported reliability gains above parent average in young bulls ranging from 2.7 to 47.6 percentage units for Holsteins, 9.6 to 29.2 percentage units for Jerseys, and 3.0 to 25.8 percentage units for Brown Swiss. In beef cattle, Garrick [30] stated that genomic prediction offers accuracies that exceed those of pedigree-based parent average of young selection candidates, and it can be equivalent to progeny tests based on up to 10 offspring. It is important to highlight that for YOUNG group, as the proportion of MS increased in the pedigree, the ssGBLUP compensated the accuracy reduction obtained with the BLUP model. In the scenario that considers all unknown sires (100% of MS), when applying the ssGBLUP model the accuracy increased from 0.05 to 0.31 for AFC and from 0.07 to 0.36 for W550, being 6 and 5 times higher for AFC and W550, respectively.

According to Olson et al. [31], the regression coefficient of TBV on EBV/GEBV is an alternative way to evaluate the genetic evaluation bias, which indicates an overestimation of the variance of genetic evaluation when it is less than 1 (inflation) and an underestimation when it is larger than 1 (deflation). In general, the regression coefficients for ALL, BULL, and FEM groups were close to 1 (Table 4), indicating that EBV predictions were less biased. For GEN and YOUNG groups, the regression coefficients were inflated as the number of unknown sires in the population increased. Large differences in regression coefficients between the BLUP and ssGBLUP models were observed for YOUNG group in all the scenarios. Biases in GEBV have been reported and discussed in several studies [8,29,32] and it could be due to the difference in scale between pedigree-based and genomic relationships, especially for young genotyped animals.

As described by Vitezica et al. [14], the comparison of average TBV and EBV/GEBV was also used to assess the bias of genetic evaluation with different proportions of multiple sires for ALL and YOUNG groups (Table 5). As expected for the scenario with 0% of MS, the average of EBV/GEBV was close to the average of TBV in ALL and YOUNG groups for both traits. As the percentage of missing pedigree increased, the BLUP and ssGBLUP models overestimated the TBV mean for both traits, mainly for young animals. However, the ssGBLUP model predicted less biased TBV mean than the BLUP model did in situations with missing pedigree.

**Table 5:** Means and standard deviations (SDs) for true breeding values (TBV) and breeding values from different traits using BLUP and ssGBLUP with different proportions of multiple sires.

	Trait	% of MS in the pedigree	BLUP	ssGBLUP
		TBV	-0.49 ± 0.50	-0.49 ± 0.50
ALL	AFC	0	-0.51 ± 0.40	-0.60 ± 0.42
		25	-0.47 ± 0.38	-0.58 ± 0.40
		50	-0.43 ± 0.36	-0.55 ± 0.38
		75	-0.39 ± 0.35	-0.50 ± 0.37
		100	-0.36 ± 0.33	-0.43 ± 0.35
		TBV	-1.30 ± 1.04	-1.30 ± 1.04
	W550	0	-1.27 ± 0.96	-1.34 ± 0.97
		25	-1.12 ± 0.92	-1.27 ± 0.93
		50	-0.99 ± 0.88	-1.14 ± 0.89
		75	-0.86 ± 0.84	-0.99 ± 0.85
		100	-0.75 ± 0.80	-0.82 ± 0.82
YOUNG	AFC	TBV	-1.01 ± 0.34	-1.01 ± 0.34
		0	-1.03 ± 0.15	-1.12 ± 0.18
		25	-0.87 ± 0.27	-0.99 ± 0.21
		50	-0.73 ± 0.30	-0.85 ± 0.23
		75	-0.46 ± 0.28	-0.58 ± 0.24
		100	-0.48 ± 0.23	-0.56 ± 0.28
		TBV	-2.54 ± 0.54	-2.54 ± 0.54
	W550	0	-2.50 ± 0.28	-2.58 ± 0.33
		25	-2.08 ± 0.62	-2.30 ± 0.41
		50	-1.70 ± 0.69	-1.95 ± 0.47
		75	-1.35 ± 0.64	-1.61 ± 0.52
		100	-1.06 ± 0.51	-1.24 ± 0.58

AFC = age at first calving; W550 = weight at 550 days; MS = multiple sires; BLUP = best linear unbiased prediction; ssGBLUP = single step genomic BLUP; ALL = all animals, YOUNG = young males without progeny

**Fig 1:**
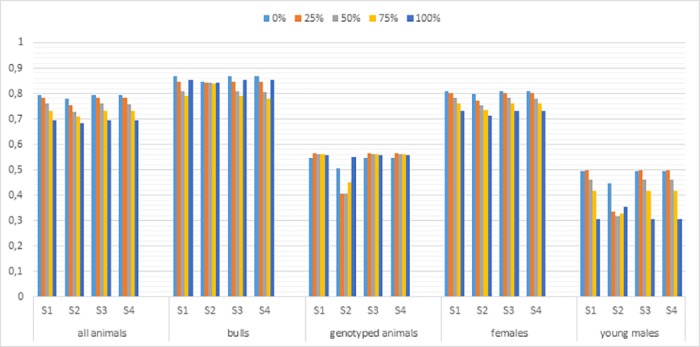
Accuracies of evaluation for AFC for the ALL, BULL, GEN, FEM, and YOUNG groups using different scaling for the genomic matrix (G) to match the numerator relationship matrix for genotyped animals (A_22_). AFC–age at first calving; S1 –scaling for all genotyped animals; S2—no scaling; S3—scaling only for animals which have known sire and dam; S4—scaling only for animals which have one known parent.

**Fig 2:**
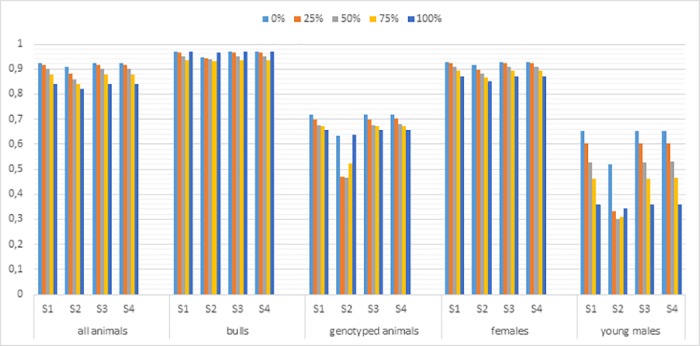
Accuracies of evaluation for W550 for the ALL, BULL, GEN, FEM, and YOUNG groups using different scaling for the genomic matrix (G) to match the numerator relationship matrix for genotyped animals (A_22_). W550—weight at 550 days; S1 –scaling for all genotyped animals; S2—no scaling; S3—scaling only for animals which have known sire and dam; S4—scaling only for animals which have one known parent.

The realized accuracy of prediction for AFC and W550 using different scaling for the **G** matrix to match to the **A**_22_ matrix is presented in Figs 1 and 2. In general, the GEBV accuracy for both traits was invariant to the scaling method applied. The GEN and YOUNG groups showed a less accurate GEBV in situations with missing pedigree and with no scaling for the **G** matrix. However, no differences between the scaling methods were observed when all the genotyped animals had unknown sires. There were no differences between the strategies for scaling the **G** matrix for ALL, BULL and FEM groups under the different scenarios with missing pedigree. These results pointed out that the uninformative part of the **A**_**22**_ matrix, i.e. genotyped animals with paternity uncertainty, did not influence the scaling of the **G** matrix. These results highlight the need to apply a G matrix in the same scale of the **A**_**22**_ matrix, especially for the evaluation of young animals in situations with missing pedigree information.

The theory for constructing the **H** matrix makes many assumptions that may not hold in practice [33]. Those assumptions include the same genetic parameters in the genotyped sample as in the whole population and the existence of complete data for all traits for which selection occurred to account for selection bias [33]. Chen et al. [12] reported that the scale of the **G** matrix influences the ranking of genotyped versus non-genotyped animals. The optimal **G** matrix should have the same average of diagonals and off-diagonals as **A**_**22**_ matrix [33]. Vitezica et al. [14] derived a formal proof and showed that a well-constructed **G** matrix with ssGBLUP model gives a more accurate and less biased GEBV than did the multistep approach.

The ssGBLUP has been used for several large-scale analyses including dairy cattle [9,34,35], beef cattle [28], pigs [11,13], and chickens [10]. These studies showed that the ssGBLUP generally was equal or more reliable than the multistep procedure and that the GEBVs were less biased. There are large differences between beef cattle compared to dairy cattle, swine, and chicken. The beef cattle production is often cited to be inferior to poultry and swine production [36], and most of the beef cattle production is in harsh environments and with low input and investment levels. In dairy cattle, a large proportion of calves in most populations are offspring of few artificial insemination sires. Thus, the lack of artificial insemination in beef contributes to poor genetic connectedness and sire identification, compromising the reliability of genetic evaluations compared to dairy cattle.

Several studies have been developed to apply the ssGBLUP model in situations with missing pedigree using unknown parent groups [1,15]. Tsuruta et al. [1] assigned UPG in mixed-model equations using the ssGBLUP model, which reduced the bias and increased the accuracy of GEBV. Misztal et al. [15] explained that potential bias could occur in genomic EBV (GEBV) using ssGBLUP with UPG. The authors reported convergence problems with iterative methods and incompatibility between the **G** and **A**_**22**_ matrices due to short or incomplete pedigrees, pedigree mistakes, incorrect assignment of genotypes, poor quality of genotypes, and the unaccounted presence of multiple/lines breeds. In our study, there were no convergence problems, even in a situation with a large proportion of missing pedigree, but it is important to emphasize that low correlations between the off-diagonal elements of the **G** and **A**_**22**_ matrices were observed due to incomplete pedigrees, mainly once the percentage of MS was higher than 50%. It is expected higher occurrence of convergence problems with real data, in which the population structure and phenotypic records are unbalanced and the model complexity is higher. Lourenco et al. [37] showed that removing old phenotypes and pedigree helped to improve convergence without decreasing accuracy for selection candidates.

According to Berry et al. [38], the development of accurate genomic evaluations in beef populations is more difficult than in dairy populations. The reasons include the presence of multiple breeds, a poor extent of phenotyping, lack of artificial insemination, and because beef systems are generally a lower-profit business that fails in adopting new technologies. The results of this study showed that when a large proportion of the pedigree is missing, the BLUP model is not reliable. However, it is possible to increase the prediction accuracy for selection candidates using the ssGBLUP model.

In many countries, several beef cattle breeding programs need to increase the availability and market of young animals with reliable genetic information in commercial herds. However, the lack of genealogy or partial pedigree information limits the reliability of the genetic evaluation in commercial herds, and, consequently, the evaluation of candidate sires. In this context, young animals with unreliable genetic evaluation are frequently discarded. Despite this study was carried out with simulated data, the results obtained with the ssGBLUP model pointed out that is possible to obtain a more reliable genetic evaluation for young animals with missing pedigree. Moreover, the breeder can have a large availability of animals for selection, increasing the selection intensity. Considering that the MS is the most common mating system in extensive beef cattle production, our results provide valuable information to support the most adequate strategy to scale the **G** matrix under paternity uncertainty scenarios, so as to increase the accuracy and decrease the bias in genetic evaluations using the ssGBLUP model. The results from this study would support breeders to reduce the risk of selecting young animals using the ssGBLUP model with missing pedigree information.

## Conclusions

Despite the ssGBLUP procedure was not developed to deal with paternity uncertainty situations, the ssGBLUP model is an appropriate alternative to obtaining more reliable and less biased breeding values in situations of missing pedigree, especially for young animals with few or no phenotypic records.

It is important to scale the **G** matrix to be compatible with the numerator relationship matrix for genotyped animals, even in situations where the latter is less informative due to the presence of missing pedigree. For accurate and unbiased genomic predictions with the ssGBLUP model, it is necessary to assure that the **G** matrix is compatible with the **A**_22_ matrix even in situations with paternity uncertainty.

## Supporting information

S1 File GEBV for simulated population (10 repetitions), considering different scenarios tested (0, 25, 50, 75 and 100% of multiple sires—MS) for W550. (RAR)

S2 File EBV for simulated population (10 repetitions), considering different scenarios tested (0, 25, 50, 75 and 100% of multiple sires—MS) for W550. (RAR)

S3 File GEBV for simulated population (10 repetitions), considering different scenarios tested (0, 25, 50, 75 and 100% of multiple sires—MS) for AFC. (RAR)

S4 File EBV for simulated population (10 repetitions), considering different scenarios tested (0, 25, 50, 75 and 100% of multiple sires—MS) for AFC. (RAR)

S5 File Data edition, variance components estimation, breeding value accuracies of all analyses. (RAR)

## References

[1] Tsuruta S, Misztal I, Lourenço DAL, Lawlor TJ. Assigning unknown parent groups to reduce bias in genomic evaluations of final score in US Holsteins. J Dairy Sci. 2014; 97: 5814–5821. doi: 10.3168/jds.2013-7821 24997668 10.3168/jds.2013-7821

[2] Henderson CR. General flexibility of linear model techniques for sire evaluation. J Dairy Sci. 1974; 57: 963–972.

[3] Kennedy BW. Bias and mean square error from ignoring genetic groups in mixed model sire evaluation. J Dairy Sci. 1981; 64: 689–697.

[4] Westell R, Quaas R, Van Vleck LD. Genetic groups in an animal model. J Dairy Sci. 1988; 71: 1310–1318.

[5] Cardoso FF, Tempelman RJ. Bayesian inference on genetic merit under uncertain paternity. Genet Sel Evol. 2003; 35: 469–487. doi: 10.1186/1297-9686-35-6-469 12939201 10.1186/1297-9686-35-6-469 PMC2697977

[6] Legarra A, Aguilar I, Misztal I. A relationship matrix including full pedigree and genomic information. J Dairy Sci. 2009; 92: 4656–4663. doi: 10.3168/jds.2009-2061 19700729 10.3168/jds.2009-2061

[7] Misztal I, Legarra A, Aguilar I. Computing procedures for genetic evaluation including phenotypic, full pedigree, and genomic information. J Dairy Sci. 2009; 92: 4648–4655. doi: 10.3168/jds.2009-2064 19700728 10.3168/jds.2009-2064

[8] Aguilar I, Misztal I, Johnson DL, Legarra A, Tsuruta S, Lawlor TJ. Hot topic: a unified approach to utilize phenotypic, full pedigree, and genomic information for genetic evaluation of Holstein final score. J Dairy Sci. 2010; 93: 743–752. doi: 10.3168/jds.2009-2730 20105546 10.3168/jds.2009-2730

[9] Tsuruta S, Aguilar I, Misztal I, Lawlor TJ. Multiple trait genomic evaluation of linear type traits using genomic and phenotypic data in US Holsteins. J Dairy Sci. 2011; 94: 4198–4204. doi: 10.3168/jds.2011-4256 21787955 10.3168/jds.2011-4256

[10] Cy Chen, Misztal I, Aguilar I, Tsuruta S, Meuwissen THE, Aggrey SE, et al Genome-wide marker-assisted selection combining all pedigree phenotypic information with genotypic data in one step: An example using broiler chickens. J Anim Sci. 2011a; 89: 23–28. 20889689 10.2527/jas.2010-3071

[11] Christensen OF, Madsen P, Nielsen B, Ostersen T, Su G. Single-step methods for genomic evaluation in pigs. Animal. 2012; 6: 1565–1571. doi: 10.1017/S1751731112000742 22717310 10.1017/S1751731112000742

[12] Chen CY, Misztal I, Aguilar I, Legarra A, Muir WM. Effect of different genomic relationship matrices on accuracy and scale. J Anim Sci. 2011b; 89: 2673–2679. 21454868 10.2527/jas.2010-3555

[13] Forni S, Aguilar I, Misztal I. Different genomic relationship matrices for single-step analysis using phenotypic, pedigree and genomic information. Genet Sel Evol. 2011; 43: 1–7. 10.1186/1297-9686-43-1 PMC3022661 21208445

[14] Vitezica ZG, Aguilar I, Misztal I, Legarra A. Bias in genomic predictions of populations under selection. Genet Res (Camb). 2011; 93: 357–366. 21767459 10.1017/S001667231100022X

[15] Misztal I, Vitezica ZG, Legarra A, Aguilar I, Swan AA. Unknown-parent groups in single-step genomic evaluation. J Anim Breed Genet. 2013; 130: 252–258. doi: 10.1111/jbg.12025 23855627 10.1111/jbg.12025

[16] Sargolzaei M, Schenkel FS. QMSim: a large-scale genome simulator for livestock. Bioinformatics. 2009; 25: 680–681. doi: 10.1093/bioinformatics/btp045 19176551 10.1093/bioinformatics/btp045

[17] Koury Filho W, Albuquerque LG, Forni S, Silva JAV, Yokoo MJ, Alencar MM. Estimativas de parâmetros genéticos para os escores visuais e suas associações com peso corporal em bovinos de corte. R. Bras. Zootec. 2010; 39: 1015–1022.

[18] Laureano MMM, Boligon AA, Costa RB, Forni S, Severo JLP, Albuquerque LG. Estimativas de herdabilidade e tendências genéticas para características de crescimento e reprodutivas em bovinos da raça Nelore. Arq. Bras. Med. Vet. Zootec. 2011; 63: 143–152.

[19] Yokoo MJ, Magnabosco CU, Rosa GJM, Lôbo RB, Albuquerque LG. Características reprodutivas e suas associações com outras características de importância econômica na raça Nelore. Arq. Bras. Med. Vet. Zootec. 2012; 64: 91–100.

[20] Brito FV, Neto JB, Sargolzaei M, Cobuci JA, Schenkel FS. Accuracy of genomic selection in simulated populations mimicking the extent of linkage disequilibrium in beef cattle. BMC Genet. 2011; 12: 80–89. doi: 10.1186/1471-2156-12-80 21933416 10.1186/1471-2156-12-80 PMC3224120

[21] Espigolan R, Baldi F, Boligon AA, Souza FR, Gordo DG, Tonussi RL, Cardoso DF, Oliveira HN, Tonhati H, Sargolzaei M, Schenkel FS, Carvalheiro R, Ferro JA, Albuquerque LG. Study of whole genome linkage disequilibrium in Nellore cattle. BMC Genomics. 2013; 14: 305 doi: 10.1186/1471-2164-14-305 23642139 10.1186/1471-2164-14-305 PMC3662636

[22] Snelling WM, Chiu R, Schein JE, Hobbs M, Abbey CA, Adelson DL, et al A physical map of the bovine genome. Genome Biol. 2007; 8:R165 doi: 10.1186/gb-2007-8-8-r165 17697342 10.1186/gb-2007-8-8-r165 PMC2374996

[23] Hayes B, Goddard ME. The distribution of the effects of genes affecting quantitative traits in livestock. Genet Sel Evol. 2001; 33: 209–229. doi: 10.1186/1297-9686-33-3-209 11403745 10.1186/1297-9686-33-3-209 PMC2705405

[24] Vanraden PM. Efficient methods to compute genomic predictions. J Dairy Sci. 2008; 91: 4414–4423. doi: 10.3168/jds.2007-0980 18946147 10.3168/jds.2007-0980

[25] Misztal I, Tsuruta S, Strabel T, Auvray B, Druet T, Lee DH. BLUPF90 and related programs (BGF90). Proceedings of the 7th World Congress on Genetics Applied to Livestock Production; 2002 August 19–23; Montpellier, France. Communication No 28–07.

[26] Aguilar I, Misztal I, Tsuruta S, Legarra A. PREGSF90 –POSTGSF90: Computational Tools for the Implementation of Single-step Genomic Selection and Genome-wide Association with Ungenotyped Individuals in BLUPF90 Programs. Proceedings of the 10th World Congress of Genetics Applied to Livestock Production; 2014; Vancouver, Canada.

[27] Nietlisbach P, Keller L, Postma E. Genetic variance components and heritability of multiallelic heterozygosity under inbreeding. Heredity. 2016; 116: 1–11. doi: 10.1038/hdy.2015.59 26174022 10.1038/hdy.2015.59 PMC4675868

[28] Lourenco DAL, Fragomeni BO, Tsuruta S, Aguilar I, Zumbach B, Hawken RJ, et al Accuracy of estimated breeding values with genomic information on males, females, or both: an example in broiler chicken. Genet Sel Evol. 2015; 47: 56 doi: 10.1186/s12711-015-0137-1 26133806 10.1186/s12711-015-0137-1 PMC4487961

[29] Wiggans GR, Vanraden PM, Cooper TA. The genomic evaluation system in the United States: Past, present, future. J Dairy Sci. 2011; 94: 3202–3211. doi: 10.3168/jds.2010-3866 21605789 10.3168/jds.2010-3866

[30] Garrick JD. The nature, scope and impact of genomic prediction in beef cattle in the United States–Review. Genet Sel Evol. 2011; 47: 1–11. 10.1186/1297-9686-43-17 PMC3107171 21569623

[31] Olson KM, Vanraden PM, Tooker ME, Cooper TA. Differences among methods to validate genomics evaluations for dairy cattle. J Dairy Sci. 2011; 94: 2613–2620. doi: 10.3168/jds.2010-3877 21524553 10.3168/jds.2010-3877

[32] Tsuruta S, Misztal I, Lawlor TJ. Short communication: Genomic evaluations of final score for US Holsteins benefit from the inclusion of genotypes on cows. J Dairy Sci. 2013; 96: 3332–3335. doi: 10.3168/jds.2012-6272 23477821 10.3168/jds.2012-6272

[33] Misztal I, Aggrey SE, Muir WM. Experiences with a single step genome evaluation. Poult Sci. 2013; 92: 2530–2534. doi: 10.3382/ps.2012-02739 23960138 10.3382/ps.2012-02739

[34] Aguilar I, Misztal I, Legarra A, Tsuruta S. Efficient computation of the genomic relationship matrix and other matrices used in single-step evaluation. J Anim Breed Genet. 2011; 128: 422–428. doi: 10.1111/j.1439-0388.2010.00912.x 22059575 10.1111/j.1439-0388.2010.00912.x

[35] Masuda Y, Misztal I, Tsuruta S, Legarra A, Aguilar I, Lourenco DA, et al Implementation of genomic recursions in single-step genomic best linear unbiased predictor for US Holsteins with a large number of genotyped animals. J Dairy Sci. 2016; 3: 1968–1974. 10.3168/jds.2015-10540 26805987

[36] Wilkinson S, Wiener P, Archibald AL, Law A, Schnabel RD, Mckay SD, et al Evaluation of approaches for identifying population informative markers from high density SNP Chips. BMC Genet. 2011; 12: 1–14. 21569514 10.1186/1471-2156-12-45 PMC3118130

[37] Lourenco DAL, Misztal I, Tsuruta S, Aguilar I, Lawlor TJ, Forni S, et al Are evaluations on Young genotyped animals benefiting from the past generations? J Dairy Sci. 2014; 97: 3930–3942. doi: 10.3168/jds.2013-7769 24679931 10.3168/jds.2013-7769

[38] Berry DP, Garcia JF, Garrick DJ. Development and implementation of genomic predictions in beef cattle. Animal Frontiers. 2016; 6: 32–38.

